# Specula for Examing Female Bladder

**Published:** 1876-04

**Authors:** 


Specula for Examining the Female Bladder.—At a meeting of the Obstetrical Society of Edinburgh, held July 14, 1875, Mr. Mathews Duncan stated, that he had recently been paying renewed attention to bladder diseases, especially chronic cystitis. He had repeatedly dilated the urethra to a large degree, and found that it relieved the cystic symptoms. Through the urethra so dilated, by Simon's vulcanite tubes, the interior of the urethra and bladder can easily be seen. In order that a thorough visual examination may be made, he now uses common glass specula, three-quarters of an inch in diameter, or larger, with internal mirror surface. They show the inside of the organs, as well as similar, but larger, specula show the vagina and cervix uteri. The whole operation of dilating and examining the bladder, exclusive of the use of chloroform, does not take more than five minutes.—*Edinburgh Medical Journal*, December, 1875.

